# Role of plasma phosphorylated neurofilament heavy chain (pNfH) in amyotrophic lateral sclerosis

**DOI:** 10.1111/jcmm.17232

**Published:** 2022-06-17

**Authors:** Chiara Zecca, Maria Teresa Dell’Abate, Giuseppe Pasculli, Rosa Capozzo, Roberta Barone, Serena Arima, Alessio Pollice, Vincenzo Brescia, Rosanna Tortelli, Giancarlo Logroscino

**Affiliations:** ^1^ Center for Neurodegenerative Diseases and the Aging Brain Department of Clinical Research in Neurology University of Bari “Aldo Moro” at “Pia Fondazione Card G. Panico” Hospital Tricase Lecce Italy; ^2^ Department of Computer Control, and Management Engineering Antonio Ruberti (DIAG) La Sapienza University Rome Italy; ^3^ Department of History, Society and Human Studies University of Salento Lecce Italy; ^4^ Department of Economics and Finance University of Bari “Aldo Moro” Lecce Italy; ^5^ Unit of Laboratory Medicine “Pia Fondazione Card.G. Panico” Hospital Lecce Italy; ^6^ Department of Basic Medicine Sciences, Neuroscience, and Sense Organs University of Bari “Aldo Moro” Bari Italy

**Keywords:** Amyotrophic Lateral Sclerosis, Biomarker, Neurofilament, Plasma

## Abstract

The phosphorylated neurofilament heavy chain (pNfH) is a promising biomarker in amyotrophic lateral sclerosis (ALS). We examined plasma pNfH concentrations in order to corroborate its role as a diagnostic and prognostic biomarker in ALS. Incident ALS cases enrolled in a population‐based registry were retrospectively selected and matched by sex and age with a cohort of healthy volunteers. Plasma pNfH levels were measured by an ELISA kit and correlated with clinical parameters. Discrimination ability of pNfH was tested using receiving operating characteristic (ROC) curves. Kaplan–Meier (KM) analysis and Cox proportional hazard models were used for survival analysis. Plasma pNfH was significantly higher in patients compared to controls. An optimal cut‐off of 39.74 pg/ml discriminated cases from controls with an elevated sensitivity and specificity. Bulbar‐onset cases had higher plasma pNfH compared to spinal onset (*p* = 0.0033). Furthermore, plasma pNfH positively correlated with disease progression rate (*r* = 0.19, *p* = 0.031). Baseline plasma pNfH did not influence survival in our cohort. Our findings confirmed the potential utility of plasma pNfH as a diagnostic biomarker in ALS. However, further studies with longitudinal data are needed to corroborate its prognostic value.

## 1 INTRODUCTION

Amyotrophic lateral sclerosis (ALS) is a neurodegenerative disorder which mainly involves the motor system, characterized by progressive degeneration of both upper and lower motor neurons.^1^ Incidence in Europe is between 2 and 3 cases per 100,000 individuals.^2^ The phenotype is highly variable, but atrophy and muscle weakness as well as fasciculations and spasticity are the most common signs. ALS usually leads to death because of respiratory failure in 2–3 years from symptom onset.^3^

To date, the diagnosis of ALS remains substantially based on clinical characteristics, including progression of symptoms over time. Electrophysiological investigations and imaging findings have a supportive role. However, due to the clinical heterogeneity of the disease and its insidious onset, the time between symptom appearance and confirmed diagnosis is still too long.^4^, ^5^ There are still no validated diagnostic biomarkers which can be expression of the underlying pathology, and can improve the diagnostic process. Currently, the most promising candidate biomarkers for ALS are neurofilaments (NFs), particularly the neurofilament light chain (NfL) and the phosphorylated neurofilament heavy chain (pNfH), which are main components of the neuronal cytoskeleton.^6^

NFs are selectively expressed in neurons and are found at the highest levels in long projection axons.^7^ After axonal injury, NFs are released into the extracellular space; thus, their concentration in CSF and/or blood reflects the degree of axonal damage.^8^

Several studies have provided evidence that NF levels are increased in patients with ALS compared to ALS mimics and healthy controls^9^, ^10^, ^11^, ^12^ and that higher NF levels are associated with faster disease progression.^10^, ^11^, ^13^

In this study, we evaluated the pNfH levels in plasma in order to corroborate its role as a diagnostic and prognostic biomarker in ALS.

## 2 METHODS

### 2.1 Study population

Incident ALS cases, diagnosed between 2011 and 2015 according to the revised El Escorial criteria,^14^ were retrospectively selected from a prospective population‐based registry in the Apulia region (SLAP). The SLAP registry was established in 1997, and the surveillance began on 1 January 1998. Cases were matched by sex and age (±3 years) with a cohort of healthy volunteers. Additional methodological information on the SLAP registry was published elsewhere.^15^ ALS patients fulfilling one of the following criteria were excluded from this study: (1) genetic ALS; (2) a concomitant diagnosis of any type of dementia including frontotemporal dementia; (3) history of major psychiatric disorders; and (4) therapy with antidepressant at the time of enrolment and/or over the previous three months (i.e. selective serotonin reuptake inhibitors, SSRIs). For the control group, the exclusion criteria were as follows: (1) presence of a first and/or a second‐degree relative with a diagnosis of ALS, frontotemporal dementia or Alzheimer's disease; (2) history of major psychiatric disorders; (3) therapy with antidepressant at the time of enrolment and/or over the previous three months (i.e. SSRIs); and (4) presence of major cardiac, renal, liver or other systemic diseases.

### 2.2 Procedures of assessment

Cases and controls underwent a detailed interview about familiar and personal history. All cases underwent a neurological examination by ALS‐expert neurologists, blinded to any biochemical result, who focused on identifying signs of upper motor neuron (UMN) and lower motor neuron (LMN) involvement and their distribution over several body regions. Diagnosis was made according to the revised El Escorial Criteria.^14^

Based on the site of symptom onset, patients were classified as (1) ‘bulbar onset’, when the onset of symptoms was in the bulbar region, or (2) ‘spinal onset’ when the onset of symptoms was in cervical, thoracic or lumbar regions. The spreading pattern of the disease was described using two variables: time to diffusion (TTD), defined as the time of symptom spreading from the onset region to a second one, and time to generalization (TTG), defined as the time of symptom spreading from the spinal or bulbar localization to both.^16^ As already described, these two clinical variables were based mostly on the personal history of the patient or on the neurological examination at baseline in a minority of cases (mostly when the neurologist detected signs, as fasciculation or spasticity in one region referred as not affected by the patient); in this case, TTD and/or TTG were considered to be present at the time of enrolment.^17^

Six clinical phenotypes were considered at the time of assessment: 1—ALS with prevalence of upper motor neuron signs, 2—ALS with prevalence of lower motor neuron signs, 3—‘flail arm’, 4—‘flail leg’, 5—bulbar ALS (patients who had not developed any spinal involvement in the first 6 months from the onset of symptoms and who had developed pyramidal signs before or after 6 months from symptoms’ onset) and 6—classical ALS.^18^

Functional status was evaluated based on the total score of the revised ALS functional rating scale (ALSFRS‐R),^19^ whereas muscular impairment was assessed using the manual muscle testing (MMT).^20^ Respiratory involvement was assessed based on forced vital capacity (FVC; percentage of the predicted value) and sniff nasal inspiratory pressure (SNIP).

The disease duration was calculated as the difference (expressed in months) between the date of assessment and the date of symptom onset. The onset‐diagnosis interval (ODI) was defined as the time‐difference (in days) between date of symptom onset and date of diagnosis. To standardize the disease progression, the progression rate was calculated as 48 minus the ALSFRS‐R score at the time of assessment and divided by disease duration from symptom onset.^11^

Other clinical information such as the presence/date of percutaneous endoscopic gastrostomy (PEG) tube placement and/or tracheostomy was collected after 24 months from the enrolment, using a telephone interview. Staging of the disease at the time of enrolment was based on the King's staging system.^21^

Mortality data were checked using a medical administrative database of Apulia region (Edotto) in which dates of death are registered. Censored date was 03 May 2019. We assumed that no ALS patient migrated outside the region during the study period.

The study was approved by the Institutional Review Board of the ‘Azienda Sanitaria Locale, Lecce’. Written informed consent was obtained from all participants or their legal next of kin if they were unable.

### 2.3 Sample collection and storage

Venous blood was drawn by venipuncture from all cases and controls; blood samples were collected in EDTA vacutainers, which were immediately centrifuged for 15 min at ~2000 g at room temperature within 1h. After centrifugation, plasma was removed, aliquoted (0.5 ml/aliquot) into screw‐cap polypropylene tubes and stored at −80°C until biochemical analyses. Samples were thawed at room temperature only once before analysis.

### 2.4 pNfH analysis

An enzyme‐linked immunosorbent assay (ELISA) was used to quantify the pNfH plasma level [Neurofilament (pNf‐H) high sensitive ELISA, REF EQ6562‐9601, For Research Use Only, Euroimmun AG], according to the manufacturer's protocol. The lower detection limit was 6 pg/ml. Briefly, in the first analysis step, the calibrators, controls and samples were diluted with monoclonal biotin‐labelled anti‐pNfH antibodies and added to microplate wells coated with polyclonal anti‐pNfH antibodies. In this process, pNfH is bound in a complex. In a second incubation, streptavidin peroxidase conjugate binds the biotin. A following incubation with substrate and chromogen promotes a colour reaction. The colour intensity is proportional to the pNfH concentration in the sample. The absorbance is then measured at 450 nm. Plasma pNfH concentrations were presented as pg/ml. Calibrators, controls and samples were measured in duplicate for each test run. The analytical performance of the assay was verified with the within‐run, between‐day and within‐laboratory precision, using two quality controls, high positive (C1) and low positive (C2), tested in triplicate in five consecutive days (CLSI EP15‐A).^22^ The within‐run CV, between‐day CV and within‐laboratory CV were 2.2%, 2.9% and 3.4%, respectively, for the C1 control (mean concentration 91.05 pg/ml), and 1.5%, 2.5% and 2.8%, respectively, for the C2 control (mean concentration 42.21 pg/ml).

### 2.5 Statistical analysis

Data are expressed as mean ±standard deviation or median ±interquartile range as appropriate, while categorical variables with absolute numbers and relative frequencies. All tests are two tailed, and a *p* < 0.05 was deemed as statistically significant.

The differences of numerical covariate between groups were evaluated with linear ANOVA and Kruskal–Wallis tests, depending on the residuals’ distributions (normal distribution assessed with the Shapiro–Wilk test) in a linear regression model and on variance homogeneity. All *p*‐values were corrected for multiple tests (Bonferroni method).

Correlations between pNfH and numerical covariates were calculated using the Spearman rank correlation coefficient.

The diagnostic performance characteristics of plasma pNfH (as a continuous variable) to discriminate between cases and controls were calculated and compared in a logistic regression analysis setting.

Age and/or gender were also tested as covariates in such context, when significant (*p* < 0.05). The area under the curve (AUC) constructed with the predicted probabilities of the logistic regression analysis and corresponding performance characteristics were reported if the AUC differed significantly from the AUC constructed with the pNfH values. The sensitivity, specificity, positive predictive value (PPV), negative predictive value (NPV) and AUC with corresponding 95% CI for plasma pNfH were calculated with receiver operating characteristic (ROC) curves. The highest Youden index was used to calculate the optimal cut‐off on a ROC analysis and consequent best serum pNfH concentration (pg/ml) discerning between ALS patients and controls.

Kaplan–Meier (KM) univariate analysis was carried out to determine the effect of plasma pNfH on survival (intended as tracheostomy and/or death events) from date of diagnosis. Log rank tests were used to test for differences among groups. Subsequently, univariable and multivariable analysis with Cox proportional hazards model was performed to estimate the hazard ratios (HR) of pNfH on survival adjusting for clinically relevant covariates. Particularly, different multivariable models were considered with age, plasma pNfH and sex as base model, consequently adding remaining clinically relevant covariates with a hierarchical strategy in order to assess the resulting adjusted hazard ratios (aHR).

All analyses were carried out using R (R development core team, Vienna, Austria) version 3.4.0.

## 3 RESULTS

### 3.1 Population characteristics

A total of 256 subjects were enrolled in the study, 128 ALS patients and 128 healthy controls matched for age (± 3 years) and sex. Demographic and clinical characteristics of the study population are listed in Table 1.

**TABLE 1:** Characteristics of study population

	ALS Patients	Controls
Number of enrolled patients: n (%)	128 (50%)	128 (50%)
Age at sampling (years): median (IQR)	64.0 (58.0 – 70.2)	66.0 (59.5 – 70.0)
Sex: *n* (%)		
Male	76 (59.4%)	76 (59.4%)
Female	52 (40.6%)	52 (40.6%)
pNfH levels (pg/ml): median (IQR)	100.7 (39.6 – 190.2)	16.0 (7.3 – 39.6)
El Escorial diagnostic categories at first evaluation: *n* (%)		
Definite	31 (24.2%)	
Probable	45 (35.2%)	
Possible	35 (27.3%)	
Suspected	17 (13.3%)	
Phenotype: *n* (%)		
ALS with prevalence of upper motor neuron signs	7 (5.8%)	
ALS with prevalence of lower motor neuron signs	30 (5.5%)	
Flail arm	1 (0.8%)	
Flail leg	0	
Bulbar	18 (15%)	
Classical ALS	64 (53.3%)	
Disease onset: *n* (%)		
Bulbar	36 (28.1%)	
Spinal	90 (70.3%)	
ALSFRS‐R at clinical evaluation^a^: median (IQR)	34 (26.8 to 40.0)	
MMT: median (IQR)	9 (8.1 – 9.5)	
FVC%: median (IQR)	91.2 (70.0 – 104.9)	
SNIP (cm H_2_O): median (IQR)	64.0 (44.0 – 81.0)	
King's Staging: *n* (%)		
2A	7 (5.5%)	
2B	16 (12.5%)	
3	80 (62.5%)	
4A	13 (10.2%)	
4B	12 (9.4%)	
Disease duration^b^ (months): median (IQR)	22 (15.0 – 33.0)	
ODI (months): median (IQR)	12 (7 – 22)	
TTD (months): median (IQR)	9 (4 – 16)	
TTG (months): median (IQR)	12 (6 – 22)	

Abbreviations

ALS, Amyotrophic lateral sclerosis; ALSFRS‐R, Amyotrophic Lateral Sclerosis Functional Rating Scale Revised; IQR, Interquartile range; ODI, onset‐diagnosis interval; TTD, time to diffusion; TTG, time to generalization.

^a^ Clinical Evaluation: time corresponding to whole blood sampling.

^b^ Disease duration: time window between symptoms onset and clinical evaluation.

### 3.2 Plasma pNfH levels in ALS and controls

**FIGURE 1:**
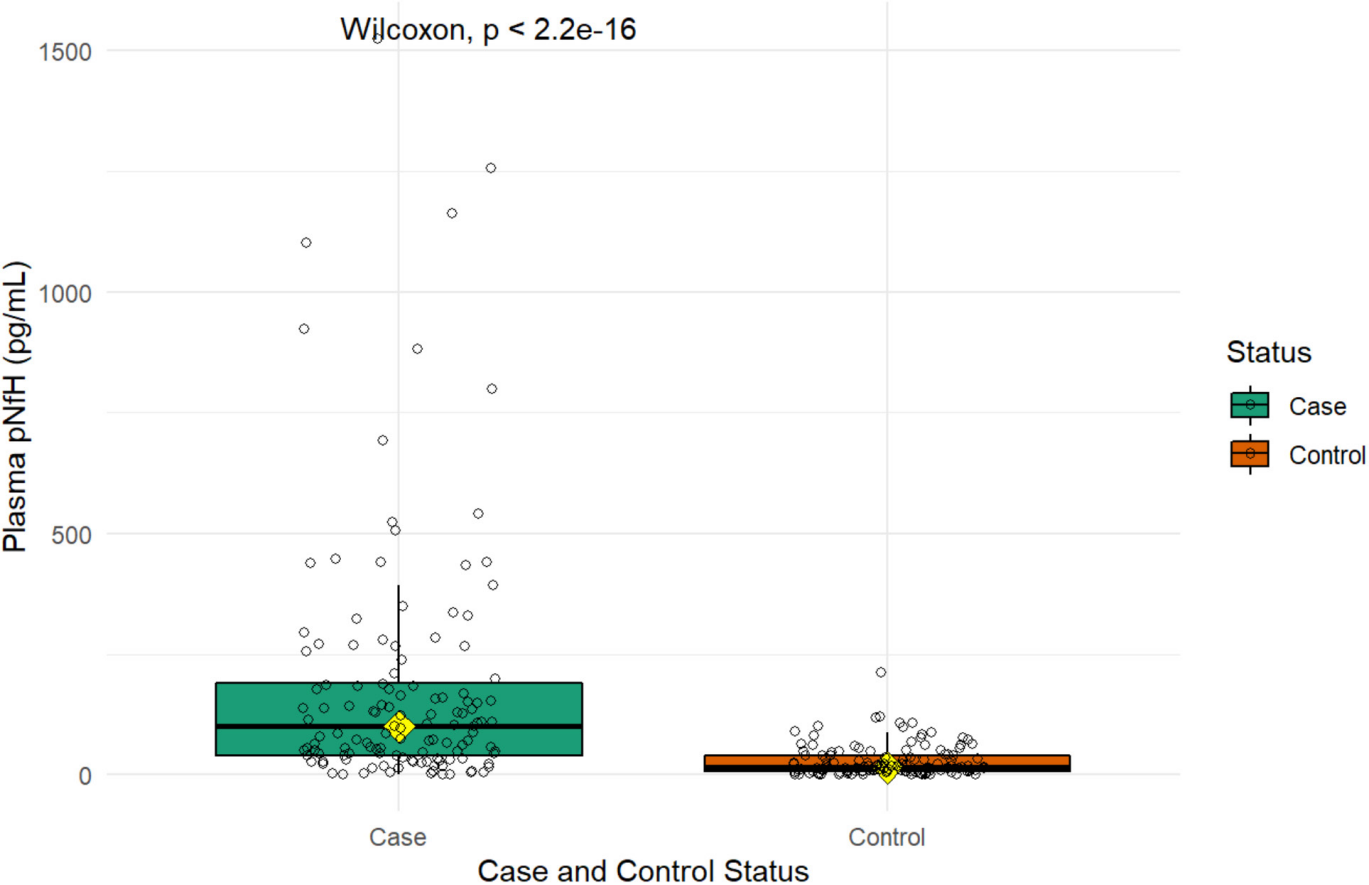
Box plot comparing plasma pNfH levels (pg/mL, y‐axis) between ALS cases and controls (x‐axis). The yellow rhombus refers to the second quartile of the boxplot (i.e. median). *p*‐value refers to the Wilcoxon rank‐sum test

The median plasma pNfH level was 100.7 pg/ml (IQR: 39.6 to 190.2) for ALS patients and 16.0 pg/ml (IQR: 7.3 to 39.6) for controls. Median values were statistically different between the two groups (*p*<0.001; Figure 1).

**FIGURE 2:**
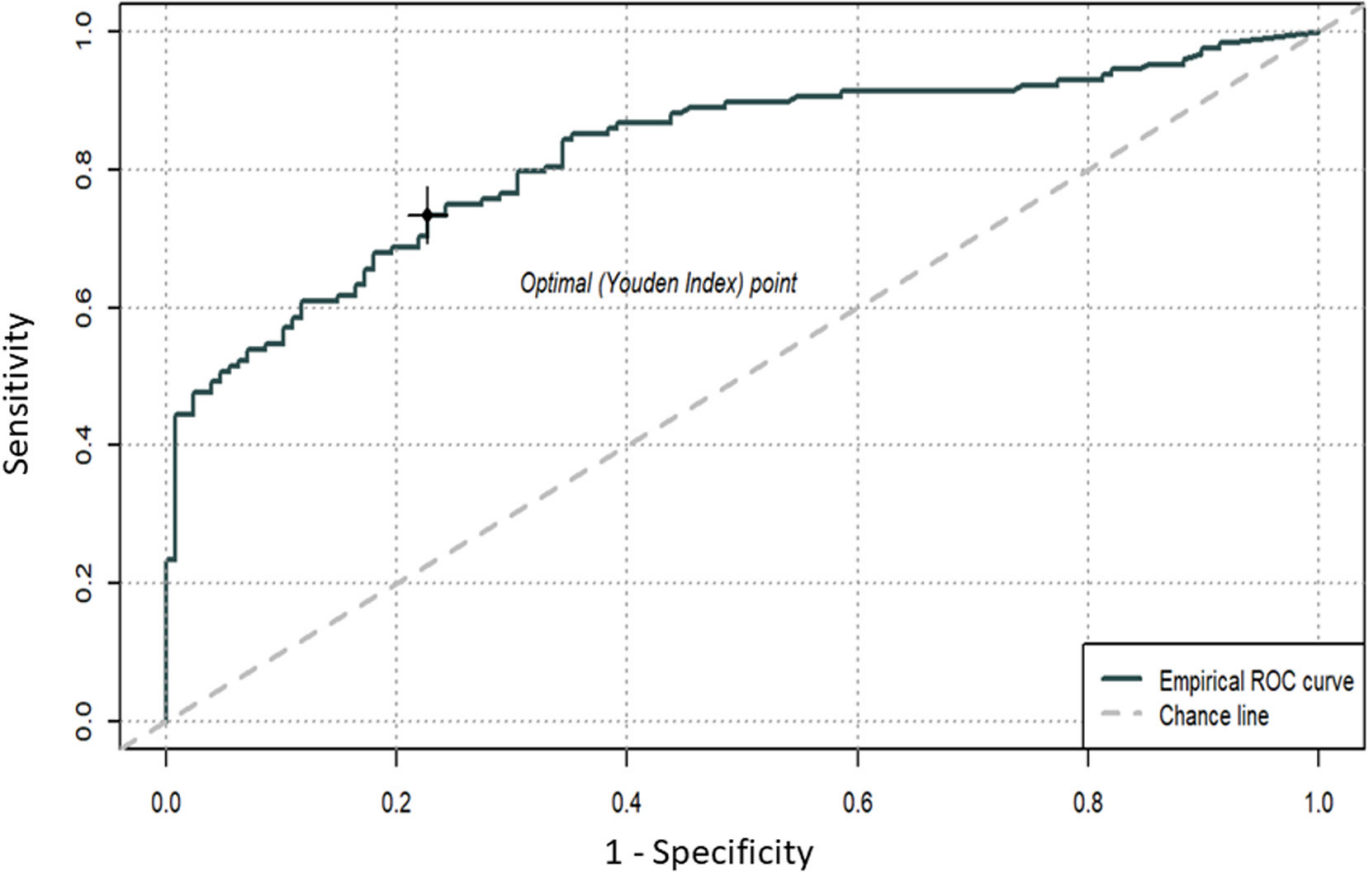
Receiver operating characteristic (ROC) curve to discriminate cases from controls, based on plasma pNfH concentration. Area under the curve (AUC) 0.82 (95% CI = 0.77–0.87). Sensitivity is shown on y‐axis and 1‐specificity on x‐axis. Youden index was used to calculate the optimal cut‐off and consequent best plasma pNfH concentration (39.74 pg/ml) discerning between ALS patients and controls

With ROC analysis, consequent to a logistic regression model with case/control status as dependent variable and biomarker as unique predictor, an optimal plasma pNfH cut‐off value of 39.74 pg/ml was calculated to discriminate between ALS cases and controls, which resulted in a sensitivity and a specificity of 75% with an area under the curve (AUC) of 0.82 (95% CI = 0.77 – 0.87). The accuracy was 73% whereas the positive predictive and the negative predictive values were 79% and 62% respectively (Figure 2).

### 3.3 Correlation with demographic characteristics and clinical parameters and group differences in cases.

In cases and controls, the correlation between plasma pNfH and age was very weak and not statistically significant (cases: *r* = −0.1, *p* = 0.26; controls: *r* = 0.057, *p* = 0.52). Plasma pNfH levels did not differ between males and females both in patients and in controls.

Restricting the analysis to cases, plasma pNfH were found significantly higher in cases with bulbar onset of the disease, compared to cases with spinal onset (*p* = 0.0033), whereas no differences were found between diagnostic categories, phenotypes at diagnosis or King's staging levels.

**FIGURE 3:**
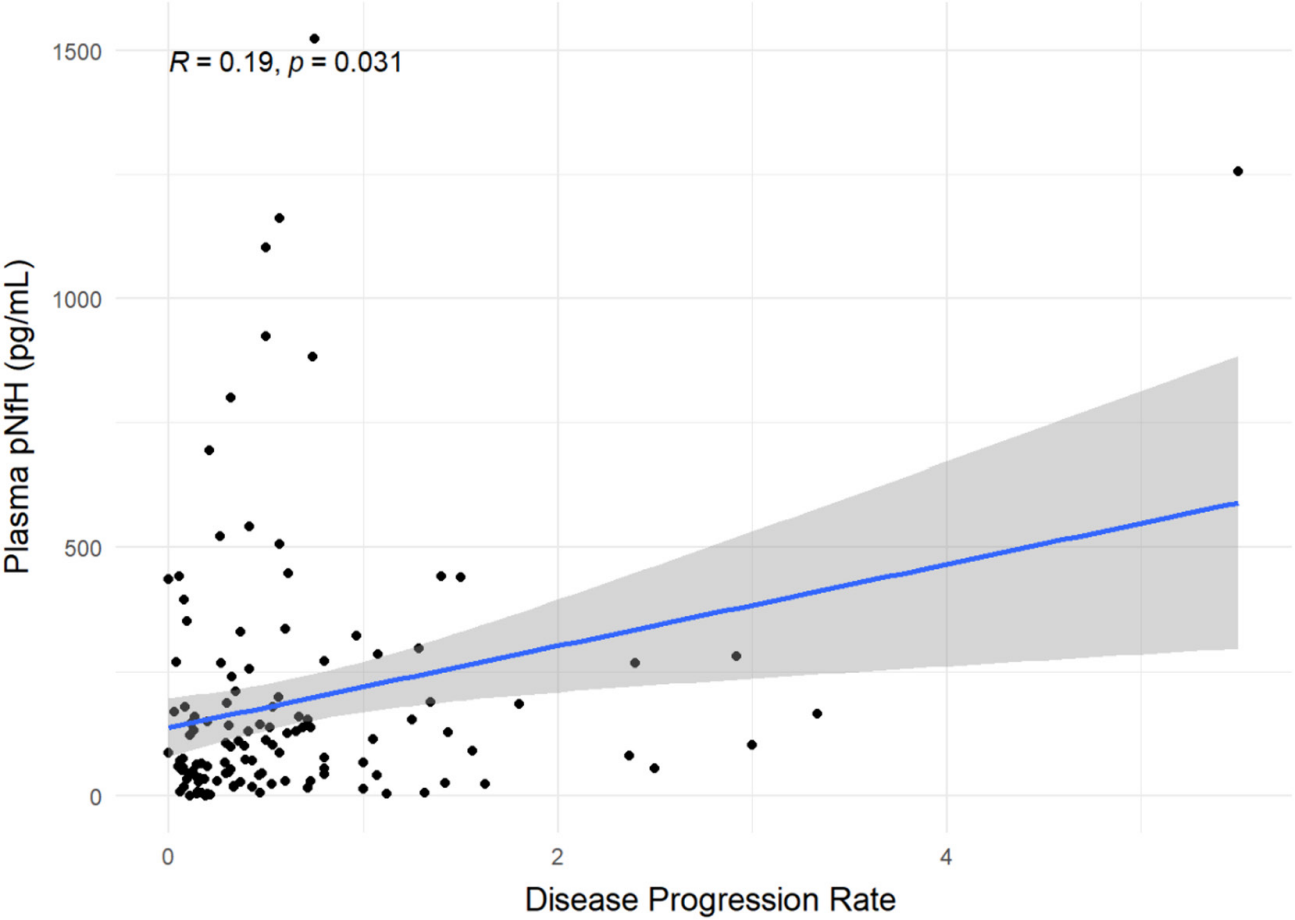
Scatter plot showing plasma pNfH levels (pg/ml, y‐axis) in ALS cases in relation to the disease progression rate (calculated as 48‐ALSFRS/disease duration, x‐axis). *p*‐value refers to Spearman's rank correlation coefficient test

Plasma pNfH positively correlated with disease progression rate. The strength of the correlation was mild–moderate, and it was statistically significant (*r* = 0.19, *p* = 0.031; Figure 3).

The strength of correlation between plasma pNfH levels and ALSFRS‐R total score was lower and not statistically significant (*r* = −0.13, *p* = 0.15). However, dividing patients into two subgroups based on the median ALSFRS‐R total score (34), mean plasma pNfH were significantly different between the two groups (*p* = 0.015). In particular, in the group with an ALSFRS‐R total score below the median the mean plasma pNfH was 244.617 (326.360 SD) pg/ml vs 142.208 (190.486 SD) pg/ml in the group with an ALSFRS‐R total score above the median (Figure S1).

The correlations of plasma pNfH levels and disease duration (*r* = −0.09, *p* = 0.33), ODI (*r* = −0.17, *p* = 0.052), TTD (*r* = −0.25, *p* = 0.358) and TTG (*r* = −0.37, *p* = 0.181) were all not statistically significant.

Eleven cases presented very high levels of plasma pNfH (>500 pg/ml). Those cases had a fast progression rate (median 0.58, range 0.26–5.5) and a short disease duration (median 7, range 2–31), although comparable with other cases with much lower pNfH plasma levels (Figure S2).

Restricting the analysis on this cluster of 11 case, the strength of the correlation between plasma pNfH levels and progression rate sharply increased, but the level of significance remained the same, probably due to the very small sample size (*r* = 0.64, *p* = 0.033; Figure S3).

### 3.4 Outcomes and survival analysis

The Kaplan–Meier product limit analysis showed no survival differences after stratifying patients according to the plasma pNfH median value (101 pg/ml; log‐rank test $\chi^{2}$ = 0.5; *p* = 0.49; Figure S4).

In univariate Cox proportional hazard (PH) regression models, higher age (HR 1.04, 95% CI = 1.01 – 1.06, *p* = 0.001), King's Stages 4a (HR 4.32, 95% CI = 1.57 – 11.886, *p* = 0.005) and 4b (HR 2.86, 95% CI 1.03 – 7.94, *p* = 0.044), faster disease progression rate (HR 1.57, 95% CI = 1.26 – 1.96, *p* < 0.001), presence of NIV (HR 2.62, 95% CI = 1.38 – 4.96, *p* = 0.003) and presence of PEG (HR 2.74, 95% CI = 1.77 – 4.26, *p* < 0.001) were independently and significantly associated with a reduced survival (defined as time from diagnosis to death/tracheostomy) in cases. On the contrary, higher ALSFRS‐R total scores at clinical evaluation were significantly associated with an increased survival (HR 0.93, 95% CI = 0.91– 0.96, *p* < 0.001).

A multivariable PH Cox regression model confirmed that age was associated with an increased adjusted HR (aHR) of 1.03 (95% CI = 1–1.06, *p* = 0.024) and that higher ALSFRS‐R scores were associated with an increased survival (aHR 0.94, 95% CI = 0.91 – 0.98, *p* = 0.001). None of the tested PH Cox regression models showed that higher plasma pNfH concentrations were independently associated with a reduced survival in cases (aHR 1, 95% CI = 1–1, *p* = 0.060).

## 4 DISCUSSION

In the present study, we measured pNfH plasma level in ALS patients and healthy controls in order to evaluate its diagnostic and prognostic significance.

We found a significant increase of plasma pNfH in cases compared to controls and we proposed an optimal cut‐off (39.74 pg/ml) for discrimination with a good sensitivity and specificity.

Our results are in line with previous studies that have consistently shown that pNfH levels are significantly elevated in serum or plasma of ALS patients compared to healthy and disease controls, suggesting that axonal damage may be detected at an early stage of the disease, even subclinical in familial ALS.^8^, ^13^, ^23^, ^24^, ^25^, ^26^ A variability of measurement of pNfH levels is present between studies. The presence of conflicting results can be explained, in part, by the high variability in the methods for pNfH measurements, not yet completely standardized; by different technologies used to measure the marker (ELISA or Simoa technology), with different diagnostic performance, but also, the study designs or the different ages of subjects enrolled can affect the comparison of results.

In the last decade, several studies, performed in larger cohorts, evaluated the diagnostic role of neurofilament subunits, above all NfL and pNfH.^6^, ^9^, ^27^ NF concentration can be measured both in cerebrospinal fluid (CSF) and in peripheral blood (plasma, serum), and although NF serum/plasma concentrations are fivefold to ten‐fold lower compared to CSF, a correlation between serum/plasma and CSF levels has been demonstrated.^13^, ^24^, ^28^ Both NfL and pNfH have been proved to be promising diagnostic biomarkers for ALS, although pNfH demonstrated a slightly higher performance than NfL in CSF in differentiating ALS from disease mimics or other neurological diseases, in terms of sensitivity and specificity (sensitivity: 78–100% pNfH vs. 85.4%–96.2% NfL; specificity: 68.8–88.0% pNfH vs. 53.5%–78.0% NfL), when measured with enzyme‐linked immunosorbent assay (ELISA).^6^, ^27^, ^29^ Moreover, pNfH in CSF performed better than serum pNfH in ALS as a diagnostic biomarker (sensitivity: 88.2% vs. 71.8%; specificity: 85.3% vs. 78.3%), and the possible formation of aggregates in serum might explain the worse diagnostic performance.^24^ It should be considered, in fact, that the precise quantification of pNfH levels in biological samples, such as serum or plasma, by immunoassay may be influenced by several issues: the possible formation of pNfH aggregates, whereby the pNfH epitope relevant for the immunoassay may be masked by the aggregate; the reduced solubility of the pNfH aggregates; the protein stability of pNfH monomers in solution, which could differ from the stability of pNfH in the aggregates. This requires in some cases the protein denaturation to overcome aggregation phenomena.^30^

We also explored the prognostic value of pNfH, and we found that baseline plasma pNfH provided little additional prognostic value beyond the effects of clinical predictors. It needs to be considered that, to date, most of the prognostic studies on NFs have been single‐centre, measuring either NfL or pNfH, performing measurement on only one biofluid, blood or CSF, and using a single assay to quantify neurofilament level, exploring either survival or functional decline, and using prospectively collected ALSFRS‐R data. The correlation between NF levels and ALSFRS‐R decline has been explored in one recent study from a large cohort of patients with ALS and related disorders which underwent careful longitudinal clinical phenotyping along with serial collection of biological samples, showing baseline serum NfL concentration, but not pNfH, predicted the future ALSFRS‐R slope.^31^

In our study, a weak/moderate but significant correlation between pNfH concentrations and the disease progression rate was found, in line with previous investigations that reported an increase of both serum and CSF pNfH in patients with a rapid disease progression.^6^, ^11^, ^13^ Shea et al have previously demonstrated that the phosphorylation of NfH slows axonal transport and its interaction with other cytoskeletal proteins, which may impact on disease course.^32^ Ganesalingam and colleagues hypothesized that a more aggressive course is associated with increased and prolonged cytoskeletal disruption within motor neurons resulting in the release of higher pNfH levels in the cerebrospinal fluid.

However, a strong prognostic role of plasma pNfH is not supported by our results. No differences in survival were found after stratifying patients according to the protein median value. Furthermore, univariate and multivariate Cox regression models, corrected for well‐characterized clinical prognostic factors, demonstrated that plasma pNfH concentrations are not independently associated with a reduced survival. This is in contrast with the study of Benatar and coworkers which showed that pNfH and NfL levels are the main predictors of patient survival.^31^ The different results may be related to the fact that Benatar and colleagues used an ultrasensitive assay to quantify both pNfH and NfL, so they dealt with a more precise measurement of the analytes’ concentration.

Even if both serum and CSF pNfH levels have been demonstrated to correlate with disease progression in previous studies, only the CSF levels were associated with the burden of upper (UMN) and lower motor neuron (LMN) involvement, assessed by clinical and neurophysiological examinations.^33^ Poesen et al. found a positive correlation between CSF NFs and the number of affected regions with both UMN and LMN involvement.^6^ This is in contrast with our results, in which plasma pNfH levels did not differ in ALS phenotypes and were not influenced by the prevalence of UMN/LMN involvement or by the number of affected regions.

In our study, plasma pNfH are somehow related to disease burden. Although the correlation between plasma pNfH with parameters of disease severity, such as the ALSFRS‐R total score, was not statistically significant, after stratifying cases according to median ALSFRS‐R, the group of patients with higher ALSFRS‐R total score had lower mean plasma pNfH levels. However, no correlation was found between pNfH levels and clinical staging in our cohort, underlying that plasma pNfH cannot be considered a good biomarker of disease burden over time. Also in other studies, NF levels did not mirror the progression through disease stages, remaining stable over the disease course except a slight decrease in later stages.^6^, ^34^

Some limitations need to be considered in this study. Firstly, healthy subjects have been used as control group. It would have been extremely useful comparing cases also to ALS‐mimic disorders for a better application of the test in clinical practice. Secondly, pNfH measurements in CSF were missing; for this reason it was not possible to correlate the plasma levels of pNfH to CSF levels. Furthermore, another limitation was the use of a classic ELISA method for plasma quantification of pNfH. The analytical sensitivity of classical ELISA assays may be not always sufficient to detect the low NF concentrations in peripheral blood. The recent introduction of new ultrasensitive assays, such as single‐molecule array (Simoa), allows detection at single‐molecule level, significantly improving analytical sensitivity,^35^ and their use in research is strongly recommended. Another limitation to consider is the relatively small sample size, which could have challenged the reliability of the more complex models; however, the sample size was adequate for the paired comparison of biomarkers’ levels between ALS and controls and in estimating the optimal cut‐off. Furthermore, the collection of samples at a single time point should also be considered: a prospective longitudinal study that obtains serial samples from ALS patients to observe the dynamic alterations of pNfH is highly recommended.

It would also be useful to have data from other biological markers. Due to the complexity of certain diseases and the diversity of biomarkers, testing a single biomarker alone is not sufficient to accurately diagnose and predict the disease. In addition, some biomarkers are not specific for some diseases or physiological changes, so it is sometimes necessary to test multiple biomarkers at the same time. Multiple biomarker testing can contribute to a more comprehensive understanding of various biological processes and disease dynamics. The recent diagnostic approaches see the concomitant measurement of different biomarkers as a tool to improve the diagnostic performance of NFs. A recent study showed that the combined measurement of NfL and TAR DNA‐binding protein‐43 (TDP‐43) on CSF had a higher diagnostic accuracy than NfL alone for ALS cases compared to age‐matched controls without neurodegenerative disease.^36^

On the contrary, the strengths of our study are as follows: 1—the matching by age and sex, which reduces the age and sex differences among the groups; 2—the use of plasma, that represents a complex but more accessible alternative to CSF for monitoring the disease; and 3—the use of data from a population‐based cohort of patients, representative of all cases from a population in a specific geographic area.

To sum up, our findings confirmed the potential utility of plasma pNfH as a diagnostic biomarker in ALS. However, further evaluations in longitudinal data are needed to corroborate its prognostic value.

## CONFLICT OF INTEREST

The authors declare that there is no conflict of interest.

## AUTHOR CONTRIBUTIONS

**Chiara Zecca:** Conceptualization (lead); Data curation (lead); Methodology (lead); Project administration (lead); Writing – original draft (lead); Writing – review & editing (lead). **Maria Teresa Dell'Abate:** Conceptualization (lead); Data curation (lead); Methodology (lead); Project administration (lead); Writing – original draft (lead); Writing – review & editing (lead). **Giuseppe Pasculli:** Formal analysis (lead); Methodology (lead). **Rosa Capozzo:** Data curation (supporting). **Roberta Barone:** Data curation (supporting). **Serena Arima:** Formal analysis (supporting); Methodology (supporting); Visualization (supporting). **Alessio Pollice:** Formal analysis (supporting); Methodology (supporting); Visualization (supporting). **Vincenzo Brescia:** Methodology (lead); Supervision (supporting). **Rosanna Tortelli:** Conceptualization (lead); Data curation (lead); Methodology (lead); Project administration (lead); Supervision (lead); Writing – original draft (lead); Writing – review & editing (lead). **Giancarlo Logroscino:** Conceptualization (lead); Funding acquisition (lead); Methodology (lead); Project administration (lead); Supervision (lead); Writing – review & editing (lead).

## Supporting information

Figure S1‐S4

## Data Availability

The data that support the findings of this study are available from the corresponding author upon reasonable request.
