# Supplementary material for: Role of plasma phosphorylated neurofilament heavy chain (pNfH) in amyotrophic lateral sclerosis
Source: J Cell Mol Med. 2022 Jun 17;26(13):3608–15. doi: 10.1111/jcmm.17232 (PMC9258711; doi:10.1111/jcmm.17232)
Supplement: Supplementary file 1 — Figure S1‐S4 [file JCMM-26-3608-s001.docx]

*
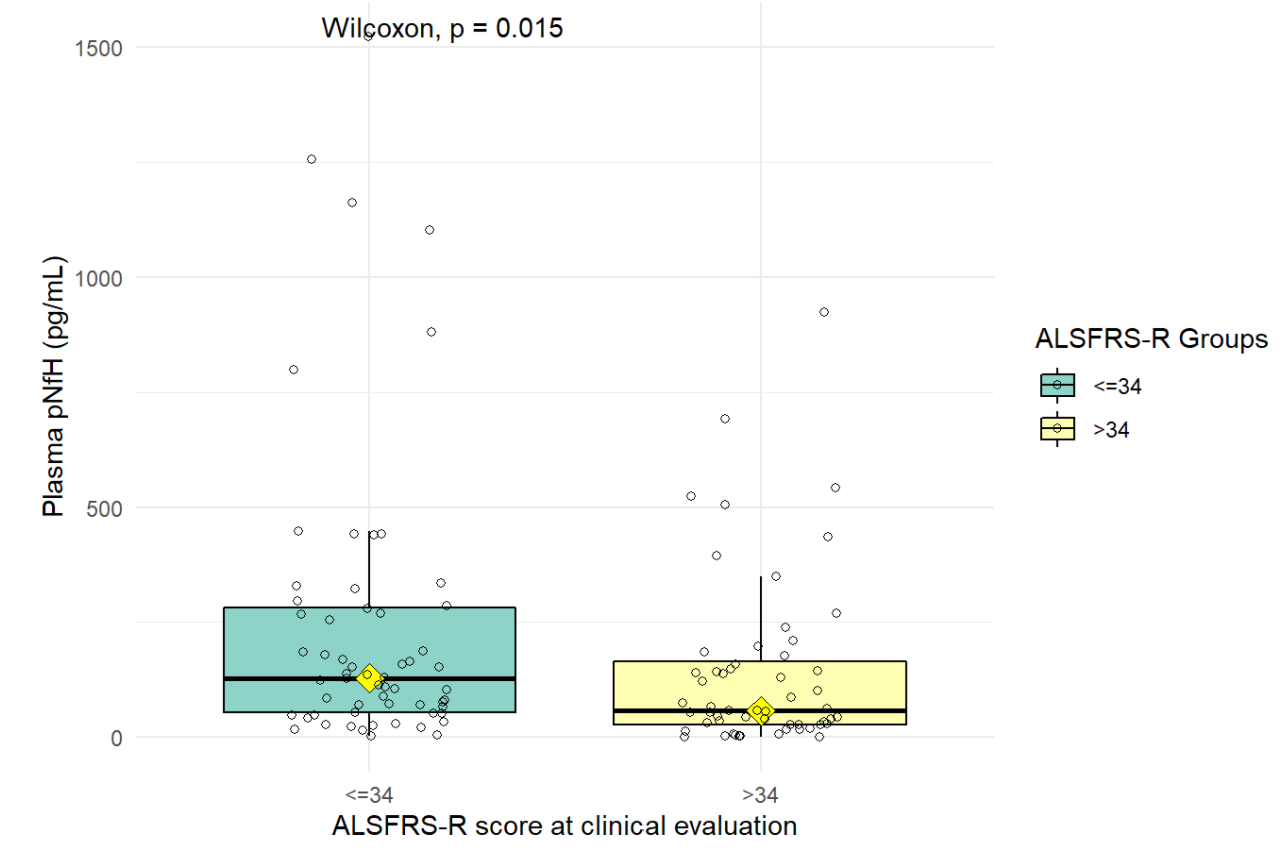
*

**Figure_1_SuppInfo**: Box plot showing plasma pNfH levels (pg/ml, y-axis) in cases stratified according to the median ALSFRS-R score at the clinical evaluation (34 as groups threshold, x-axis). The yellow rhombus refers to the second quartile of the boxplot (i.e. median). p-value refers to the Wilcoxon rank-sum Test.


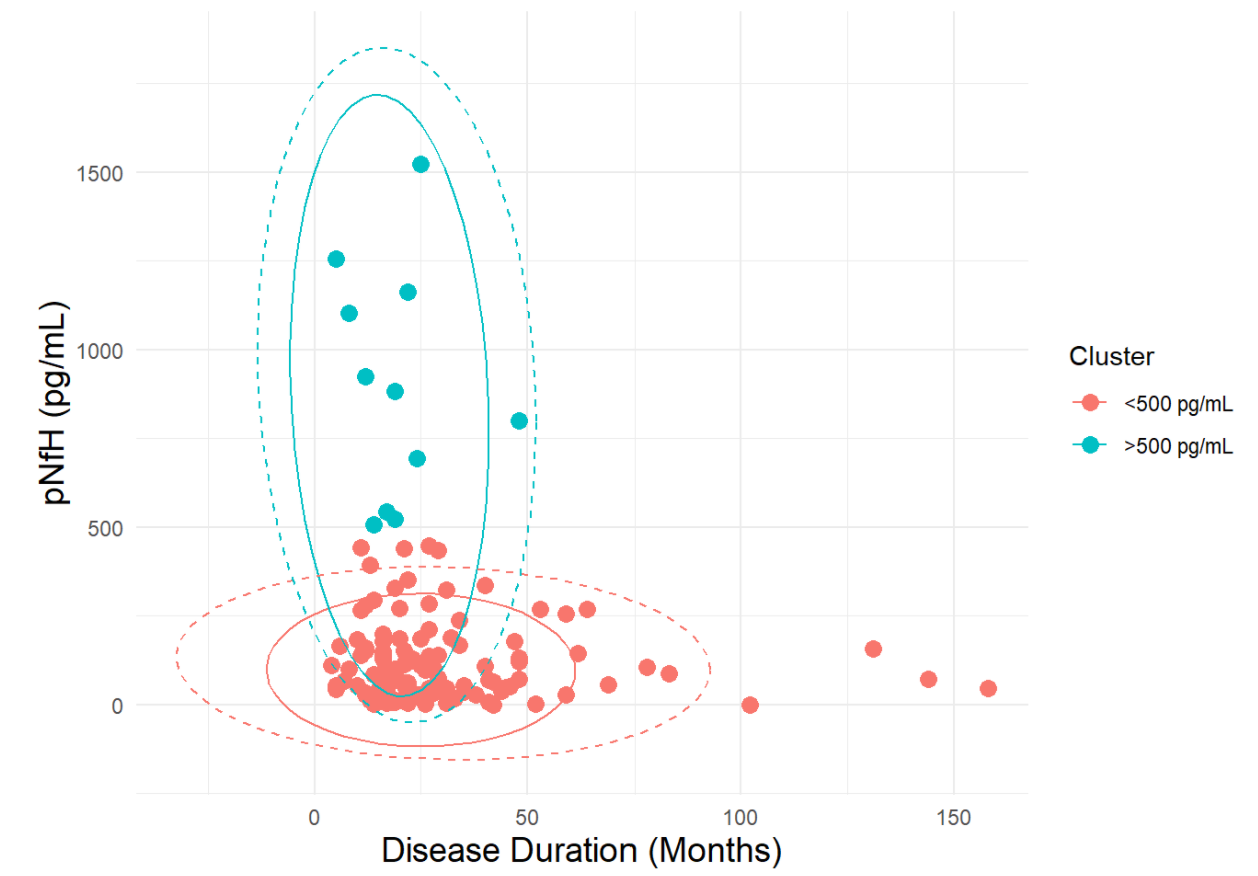


**Figure_2_SuppInfo:** Scatter plot showing plasma pNfH levels (pg/ml) in ALS cases in relation to disease duration (months) highlighting clusters of patients with > 500 pg/ml of plasma pNfH. Continuous ellipsis assuming multivariate t-distribution and dashed ellipsis assuming multivariate normal distribution are also shown in order to better highlight cluster distributions along their elliptic tolerance intervals within the plot.


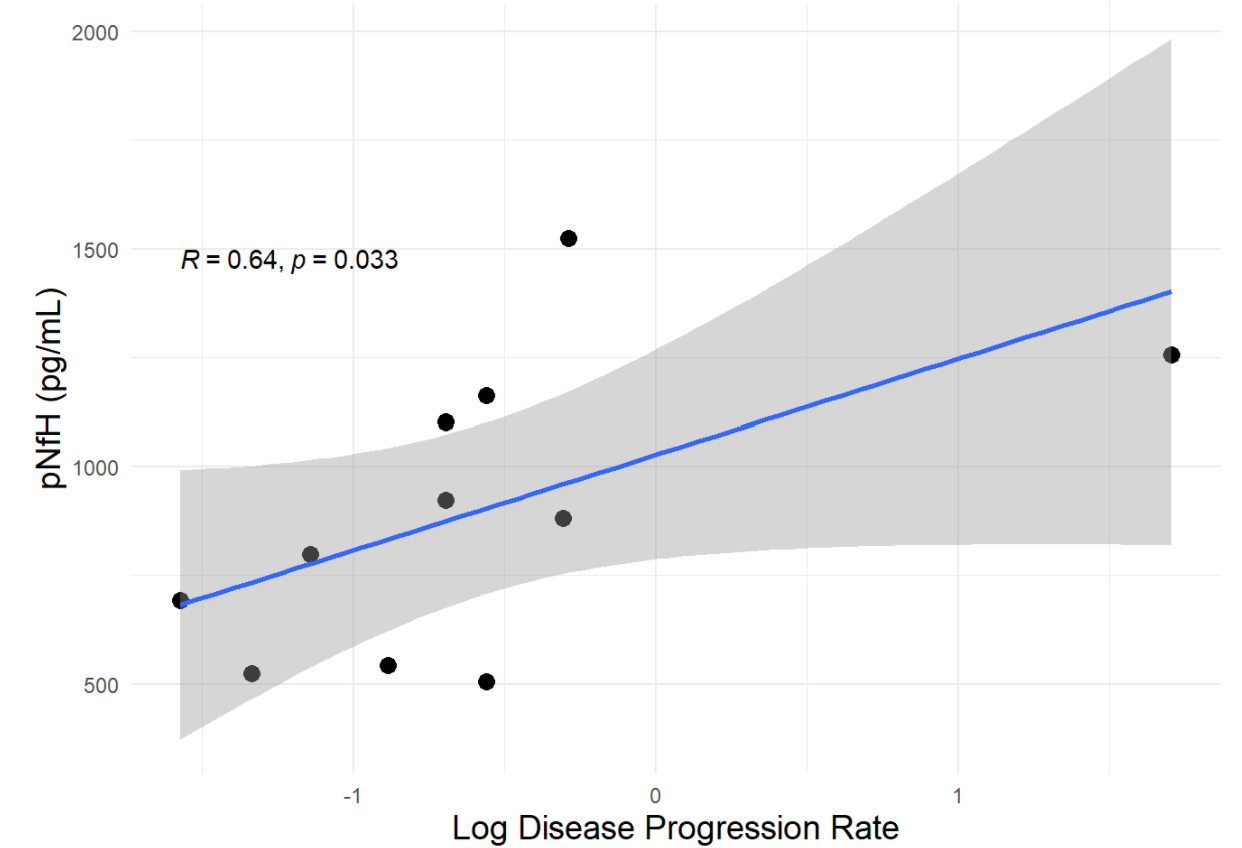


**Figure_3_SuppInfo:** Scatter plot showing plasma pNfH levels (pg/ml) in relation to the (natural log) of disease progression rate (calculated as 48-ALSFRS/Disease Duration) in the cluster of cases with pNfH levels > 500 pg/ml. p-value refers to Spearman's rank correlation coefficient test.

**
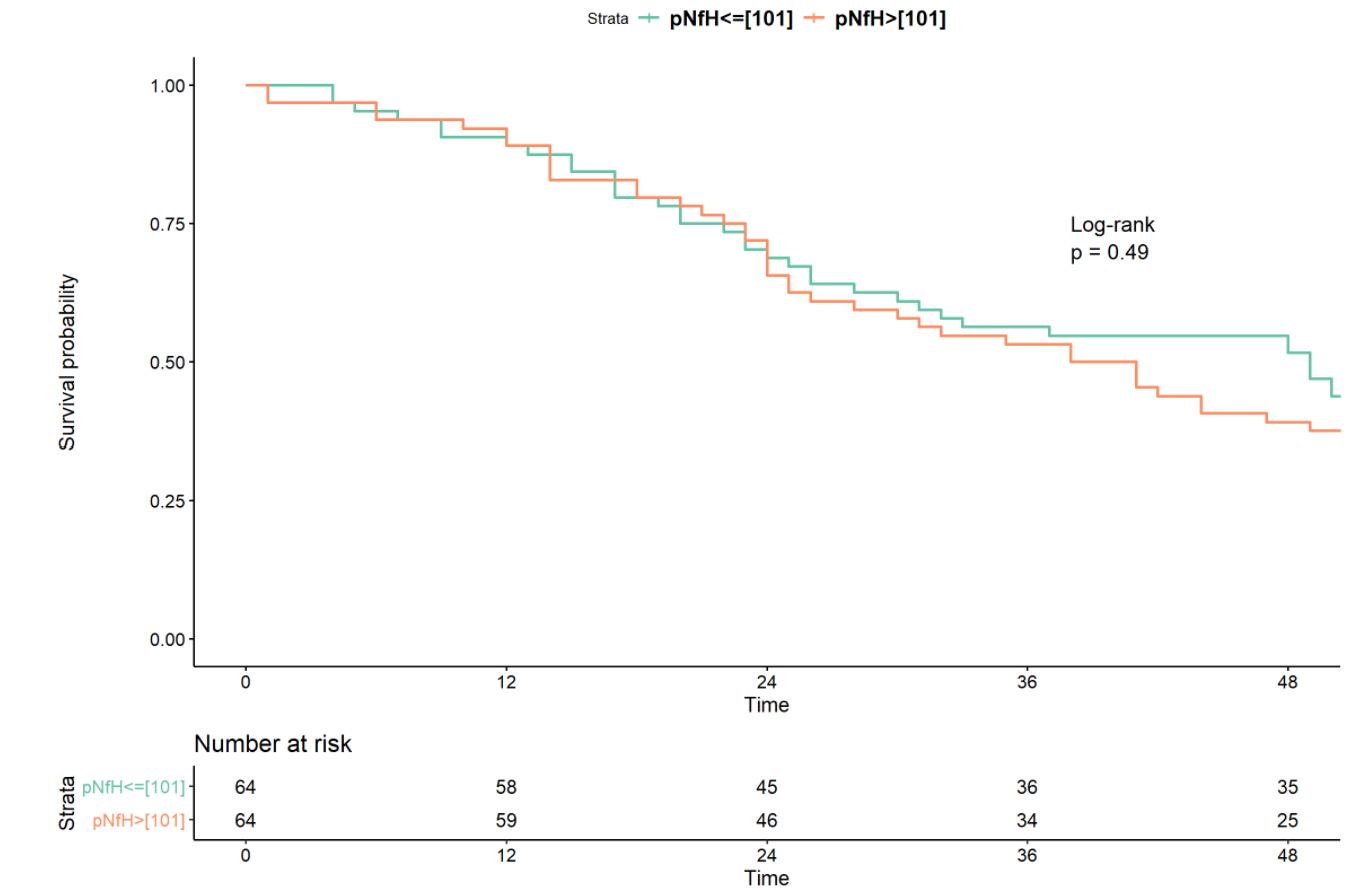
**

**Figure_4_SuppInfo:** Kaplan-Meier survival curves in patients with ALS stratified according to the median plasma pNfH level (101 pg/mL) (Log-rank test chi-squared=0.5; p=0.49).
